# Unique Findings of Sickle Cell Retinopathy in a Patient with Hemoglobin SE Disease

**DOI:** 10.18502/jovr.v20.14699

**Published:** 2025-06-18

**Authors:** Corey Lacher, Aliya C. Roginiel, Elmira Baghdasaryan, Alexander A. Svoronos, Philip J. Ferrone, Isha Cheela

**Affiliations:** ^1^Department of Ophthalmology and Visual Science, Rutgers New Jersey Medical School, Newark, NJ, USA; ^2^Shiley Eye Institute, University of California San Diego, San Diego, CA, USA; ^3^Northwell Health Eye Institute, New Hyde Park, NY, USA; ^4^Department of Ophthalmology, Manhattan Eye, Ear and Throat Hospital, New York, NY, USA; ^5^Vitreoretinal Consultants of New York, Great Neck, NY, USA; ^6^Zucker School of Medicine at Hofstra/Northwell, Hempstead, NY, USA

**Keywords:** African American, Pediatric Screening, Retinopathy, Sickle Cell Disease

## Abstract

**Purpose:**

To report the second documented case of spontaneous sickle cell retinopathy due to hemoglobin SE disease, and the third in association with this condition overall.

**Case Report:**

An asymptomatic 19-year-old African American woman with hemoglobin SE disease and no other significant past medical history presented for a routine eye exam. Fundoscopy revealed two sunburst lesions in the temporal periphery of her right eye and one such lesion in the temporal periphery of her left eye. No definitive signs of neovascularization were detected on fluorescein angiography, although multiple areas of abnormal vasculature and distal non-perfusion were observed.

**Conclusion:**

Spontaneous peripheral retinopathy can develop at an early age in hemoglobin SE disease. Given the risk for complications, pediatric screening with regular fundoscopic examination may benefit such patients.

##  INTRODUCTION

Unlike the more prevalent sickle cell disease variants hemoglobin SS (HbSS) and hemoglobin SC (HbSC), hemoglobin SE (HbSE) disease is poorly characterized and infrequently diagnosed, with less than a few dozen known cases. The hemoglobin E allele is mainly found in people of Southeast Asian rather than African descent,^[1]^ and HbSE disease is a double heterozygous condition of both HbE and HbS alleles.

Sickle cell retinopathy (SCR) is a known complication of many sickle cell disease variants, with the highest association with HbSC disease. SCR can lead to visual impairment, vitreous hemorrhage, and retinal detachment.^[2]^ While HbSE patients can have systemic manifestations, it is often difficult to detect, as patients present with milder symptoms.^[3]^ Sickle cell disease can affect both the anterior and posterior segments of the eye, although non-proliferative and proliferative changes of the retina are the most common findings.^[1]^ To date, only two cases of SCR in patients with HbSE disease have been reported, one of which was instigated by intraocular pressure (IOP) elevation in the setting of traumatic hyphema, while the other developed proliferative disease spontaneously.^[4,5]^ In this report, we document the case of a young woman with HbSE disease, otherwise asymptomatic and without comorbidities, presenting to the outpatient office with unique retinal findings of SCR.

##  CASE PRESENTATION

A 19-year-old African American woman with HbSE disease presented with a chief complaint of floaters in both eyes. The patient had a known diagnosis of HbSE disease on newborn screening. Based on a genetics consultation, her mother, father, and brother have known sickle cell traits. The mother is a carrier of HbE, the father and brother are carriers of HbS. Interestingly, the brother has a daughter with HbSE. Other than one hospitalization for pneumococcal sepsis when she was seven years old, the patient had been healthy, and not on any medications. High-performance liquid chromatography (HPLC) with capillary electrophoresis, which can distinguish hemoglobin E from hemoglobin A ${}_{2}$ ^[6]^ had revealed 61.4% hemoglobin S, 26.9% hemoglobin E, 4.7% hemoglobin A ${}_{2}$ , and 7.0% hemoglobin F in our patient.

**Figure 1:**
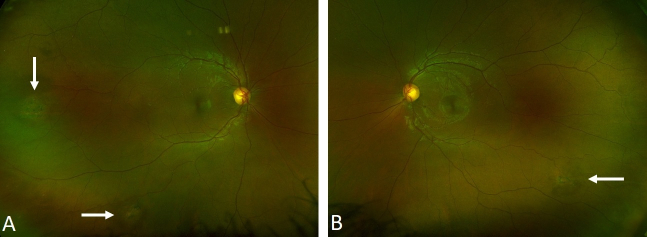
Color fundus photographs of (A) right eye and (B) left eye revealing peripheral sunburst lesions (white arrows).

**Figure 2:**
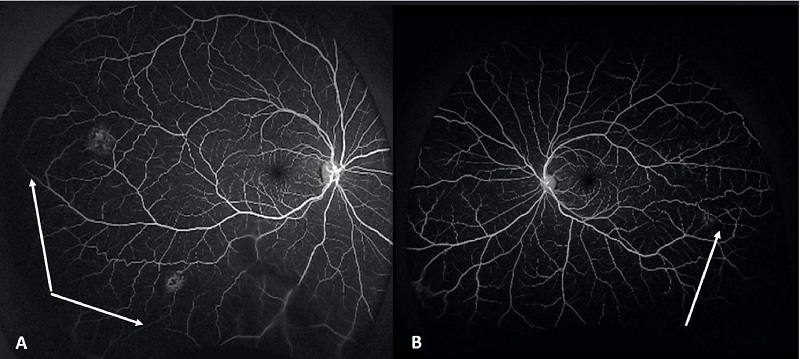
Fluorescein angiography of (A) right eye and (B) left eye demonstrating nonperfusion distal to the sunburst lesions (white arrows).

On examination, the best corrected visual acuity was 20/20 in the right eye and 20/15 in the left eye, with normal IOP and unremarkable anterior segment exam. Posterior segment exam [Figure 1] was remarkable for two sunburst pattern lesions in the right eye and one in the left. Fluorescein angiography showed areas of temporal non-perfusion distal to the sunburst lesions in both eyes [Figure 2]. Our patient was followed by an ophthalmologist throughout 2.5 years with stable ocular examination findings without any episodes of vaso-occlusive crisis and blood transfusions.

##  DISCUSSION

SCR is a common ocular manifestation in patients with sickle cell disease, with a prevalence of 42% in the second decade of life.^[7]^ Sickling occurs when physiologic insults expose hydrophobic motifs on hemoglobin molecules, leading to the creation of long hemoglobin fibers within erythrocytes.^[8]^ These fibers cause erythrocyte rigidity and damage, leading to vaso-occlusion, vascular endothelial dysfunction, and inflammation.^[8]^ Damage to the retinal vessels can lead to both non-proliferative and proliferative changes to the retina, as characterized by the Goldberg classification.^[9]^ Signs of non-proliferative retina changes can be represented as “salmon patches” – round or oval retinal hemorrhages from superficial blood vessels. Migration and proliferation of the retinal pigment epithelium most likely occur in response to those hemorrhages and lead to the development of black sunburst spots. Proliferative changes are described mainly by the development of peripheral retinal neovascularization, which can be missed only if imaged with standard fluorescein angiography. Of these complications, the most serious are vitreous hemorrhage and retinal detachment, usually found in neovascular zones.^[10]^

Two cases of HbSE retinopathy have previously been reported. The first one documented retinal vascular occlusion in a 23-year-old Omani Arab man attributed to IOP elevation from traumatic hyphema and osmotically induced hyper-viscosity from glycerin and mannitol.^[4]^ The second case was a 56-year-old African American man with findings of sea-fan peripheral neovascularization, necessitating sectoral laser photocoagulation.^[5]^ Our case is similar to the latter as the retinal lesions arose spontaneously in an African American and were detected without ocular symptoms.

Unlike the previous reported case of spontaneous proliferative SCR in HbSE disease,^[5]^ our patient did not present with definitive signs of neovascularization. However, sunburst lesions, areas of retinal pigment epithelial hypertrophy classically seen in SCR, were visualized in both eyes. Furthermore, there were no signs of peripheral neovascularization or vessel leakage of fluorescein dye during fluorescein angiography. Thus, our patient demonstrated early stages of non-proliferative retinopathy. This could be due to a lack of comorbidities, younger age, or simply earlier detection of the condition. Interestingly, as with the previous case, our patient was also of African descent, with no known Asian ancestry, which is unusual for carriers of the hemoglobin E allele. While only two cases have been documented, this raises the question of whether spontaneous retinopathy is more prevalent in HbSE patients of African descent.

Our report is the second to document spontaneous SCR secondary to HbSE disease. As HbSE disease tends to be clinically milder than HbSS and HbSC disease, it is unclear what percentage of these patients develop SCR. Although both our patient and the patient from the previously documented case^[5]^ were asymptomatic, they both exhibited findings consistent with sickling and occlusion of the retinal vasculature. Our case report is clinically relevant as it describes the development of retinal lesions in an asymptomatic patient at a younger age, which may result in the development of proliferative retinopathy. Therefore, due to the potentially sight-threatening complications associated with SCR, we recommend annual pediatric screening for HbSE patients and regular follow-up with a retina specialist for known retinal disease. In summary, this is a case report about ocular manifestations of a relatively rare inherited blood disorder. Larger cohort studies are warranted to devise a proper screening plan using emerging imaging technology along with genetic analysis, as the increasing rate of migrations and interracial marriages is expected to increase the HBSE cases in general.

##  Financial Support and Sponsorship

None.

##  Conflicts of Interest

None.

## References

[B1] Mishra P, Pati HP, Chatterjee T, Dixit A, Choudhary DR, Srinivas MU, et al (2005). Hb SE disease: A clinico-hematological profile. Ann Hematol.

[B2] Abdalla Elsayed ME, Mura M, Al Dhibi H, Schellini S, Malik R, Kozak I, et al (2019). Sickle cell retinopathy. A focused review Graefes Arch Clin Exp Ophthalmol.

[B3] Masiello D, Heeney MM, Adewoye AH, Eung SH, Luo HY, Steinberg MH, et al (2007). Hemoglobin SE disease - A concise review. Am J Hematol.

[B4] Ganesh A, Ai-Habsi NS, Ai-Alawi FK, Mitra S, Eriksson A, Venugopalan P (2000). Traumatic hyphaema and sickle cell retinopathy in a patient with sickle cell-haemoglobin E (HbSE) disease. Eye.

[B5] Baciu P, Yang C, Fantin A, Darnley-Fisch D, Desai U (2014). First reported case of proliferative retinopathy in hemoglobin SE disease. Case Rep Ophthalmol Med.

[B6] Mais DD, Gulbranson RD, Keren DF (2009). The range of hemoglobin A(2) in hemoglobin E heterozygotes as determined by capillary electrophoresis. Am J Clin Pathol.

[B7] de Melo MB (2014). An eye on sickle cell retinopathy. Rev Bras Hematol Hemoter.

[B8] Giese K, Jerome Clay EL, Roland B (2020). Scott, MD. Sickle cell symposium J Natl Med Assoc.

[B9] Goldberg MF (1971). Classification and pathogenesis of proliferative sickle retinopathy. Am J Ophthalmol.

[B10] Wang M, Hussnain SA, Chen RW (2019). The role of retinal imaging in sickle cell retinopathy: A review. Int Ophthalmol Clin.

